# Carvedilol Phenocopies PGC-1α Overexpression to Alleviate Oxidative Stress, Mitochondrial Dysfunction and Prevent Doxorubicin-Induced Toxicity in Human iPSC-Derived Cardiomyocytes

**DOI:** 10.3390/antiox12081585

**Published:** 2023-08-09

**Authors:** Nnamdi Uche, Qiang Dai, Shuping Lai, Kurt Kolander, Mai Thao, Elizabeth Schibly, Xavier Sendaydiego, Jacek Zielonka, Ivor J. Benjamin

**Affiliations:** 1 Cardiovascular Center, Department of Physiology, Medical College of Wisconsin, 8701 W Watertown Plank Road, Milwaukee, WI 53226, USA; nuche@mcw.edu; 2 Cardiovascular Center, Division of Cardiovascular Medicine, Department of Medicine, Medical College of Wisconsin, 8701 W Watertown Plank Road, Milwaukee, WI 53226, USA; qdai@mcw.edu (Q.D.); slai@mcw.edu (S.L.); kurt.kolander@cuw.edu (K.K.); maitho2047@gmail.com (M.T.); eschibly@mcw.edu (E.S.); xgsenday@uw.edu (X.S.); 3 Free Radical Laboratory, Department of Biophysics, Medical College of Wisconsin, 8701 W Watertown Plank Road, Milwaukee, WI 53226, USA; jzielonk@mcw.edu

**Keywords:** redox homeostasis, bioenergetics, doxorubicin, cardiomyocytes, carvedilol, prophylaxis, preclinical, human iPSCs

## Abstract

Doxorubicin (DOX), one of the most effective and widely used anticancer drugs, has the major limitation of cancer treatment-related cardiotoxicity (CTRTOX) in the clinic. Reactive oxygen species (ROS) generation and mitochondrial dysfunction are well-known consequences of DOX-induced injury to cardiomyocytes. This study aimed to explore the mitochondrial functional consequences and associated mechanisms of pretreatment with carvedilol, a ß-blocking agent known to exert protection against DOX toxicity. When disease modeling was performed using cultured rat cardiac muscle cells (H9c2 cells) and human iPSC-derived cardiomyocytes (iPSC-CMs), we found that prophylactic carvedilol mitigated not only the DOX-induced suppression of mitochondrial function but that the mitochondrial functional readout of carvedilol-pretreated cells mimicked the readout of cells overexpressing the major regulator of mitochondrial biogenesis, PGC-1α. Carvedilol pretreatment reduces mitochondrial oxidants, decreases cell death in both H9c2 cells and human iPSC-CM and maintains the cellular ‘redox poise’ as determined by sustained expression of the redox sensor Keap1 and prevention of DOX-induced Nrf2 nuclear translocation. These results indicate that, in addition to the already known ROS-scavenging effects, carvedilol has a hitherto unrecognized pro-reducing property against the oxidizing conditions induced by DOX treatment, the sequalae of DOX-induced mitochondrial dysfunction and compromised cell viability. The novel findings of our preclinical studies suggest future trial design of carvedilol prophylaxis, such as prescreening for redox state, might be an alternative strategy for preventing oxidative stress writ large in lieu of the current lack of clinical evidence for ROS-scavenging agents.

## 1. Introduction

For decades, the anthracycline antibiotic doxorubicin (DOX) has been used as an effective chemotherapy agent for a wide range of cancers [1,2]. However, cancer treatment-related cardiotoxicity (CTRTOX) from chemotherapy, including the anthracycline DOX, is a well-established dose-dependent complication that has markedly limited its clinical application and restricted the eligibility of many patients from receiving effective treatment as an attempt to spare the deleterious consequences on the cardiovascular system. Such untimely and premature discontinuation of DOX therapy has inevitably and regrettably not only resulted in poorer overall outcomes but has negatively affected the quality of life in survivors [3].

Therefore, considerable investments have been made to identify and evaluate the best prospects for cancer patients to utilize preventative strategies against CTRTOX. The DOX-induced cardiotoxicity (DIC) experienced by patients ranges from asymptomatic reductions in the left ventricular ejection fraction to cardiomyopathy predisposing to congestive heart failure [4,5]. Research over recent decades has sought to better understand the mechanistic implications and the consensus has arisen around DOX-induced generation of free radicals and damage to the cellular components including DNA [6,7]. The postmitotic property of cardiomyocytes makes the heart particularly vulnerable and prone to oxidation [8], owing to the high mitochondrial volume of cardiomyocytes to match the heart’s energy demands [9,10], of the effects on cardiolipin, a phospholipid known to complex irreversibly with DOX [11,12], as well as the accumulation of mitochondrial DNA (mtDNA) mutations [13]. DOX therapy is also known to result in damage to the cardiac vasculature via the killing of cells including vascular endothelial cells and pericytes. The most ideal cardioprotective effort would leave the antineoplastic effect of the anthracycline unaltered. Because the susceptibility of rapidly proliferating cancer cells remains the principal and desirable mechanism of the efficacy of DOX, a secondary consideration is related to DNA damage as a strategy for cardioprotection [14]. Given the intersectionality between the mechanisms of cancer cell killing and cardiomyocyte toxicity, the translational discipline of cardio-oncology seeks to fully realize the current elusive goal for the protection of the heart while simultaneously leaving the antineoplastic efficacy of cancer treatment unaltered.

Among the options currently available, cancer patients will either face CTRTOX during the completion of effective cancer treatment or ultimately receive suboptimal treatment for their cancer to avert the risk of the aforementioned negative cardiovascular outcomes. However, recent clinical trials for primary prevention of cardiotoxicity by Food and Drug Administration (FDA)-approved drugs have yielded negative and controversial results [15,16,17]. For example, dexrazoxane has been solely approved by the United States FDA as a cardioprotective agent for combination therapy with DOX in cancer treatment. While the mechanism for cardioprotection includes the prevention of free radical generation via intracellular iron chelation [12], dexrazoxane use had been controversial [18] due to concerns about adverse secondary malignancies [19] and hematological events [20]. In the United States and some European countries, its use has been restricted to adult anthracycline patients with either advanced or metastatic cancer [21].

The clinical demand and translational research to identify new agents and/or preventative strategies that offer cardioprotection against DIC are, therefore, still warranted and are being actively investigated. Substantial work in recent decades has considered the mechanistic underpinnings of DIC to generate new targets for prevention. Mitochondrial dysfunction including alterations to the electron transport chain (ETC)/oxidative phosphorylation system (OXPHOS) [22,23,24], damage to mitochondrial DNA [22], diminished fatty acid transport [25] and oxidative stress [26] can result in devasting consequences to the contractile units of the heart including the cardiomyocyte. Antioxidant therapy has failed to give a substantial contribution in terms of cardioprotection [27]. Recent studies have provided proof of concept for the notion that mitochondrial dysfunction, a precursor of impaired cardiac function and heart failure, might be reversed by preventive strategies.

In a recent randomized control trial, patients who were enrolled in an arm of individualized and structured exercise therapy for 12 months markedly improved cardiopulmonary fitness using the volume of peak oxygen uptake (VO_2_ peak), cardiac function and reduced functional disability compared with usual care arm receiving cardiotoxic chemotherapy [28]. While citing the strengths of these positive findings and real-world impact, the investigators acknowledged the potential for selection bias by including better-resourced (e.g., 75% > tertiary education) and motivated individuals.

Carvedilol is a beta (β)-blocker commonly prescribed for the treatment of congestive heart failure and hypertension [29,30]. Multiple pleiotropic actions of this drug have been identified including antioxidant, vasodilatory, anti-inflammatory and mitochondrial protective properties [31]. A meta-analysis of eight randomized controlled trials (>600 total patients), which evaluated the efficacy of carvedilol for the primary prevention of anthracycline-induced cardiotoxicity, has revealed that prophylactic administration of carvedilol in anthracycline-treated cancer patients reduces the early onset of left ventricular (LV) dysfunction [32]. Carvedilol treatment had also been shown to significantly reduce circulating troponin levels, a marker of cardiac injury, and diastolic dysfunction in patients who received carvedilol during chemotherapy treatment [33]. While the cardio-protective effects of carvedilol against CTRTOX have increasingly been appreciated, the exact mechanism of this protection remains to be elucidated. Though ROS-scavenging [34] and ROS-suppressive effects [35] have been attributed to carvedilol, we tested the alternative hypothesis that the cardioprotective effects of carvedilol are mechanistically linked to improving cardiometabolic fitness at the level of mitochondrial function to ameliorate and/or prevent DOX-induced oxidative stress and cell death using both H9c2 cells and human induced pluripotent stem cell-derived cardiomyocyte (iPSC-CM) models. Our findings indicate for the first time that carvedilol pretreatment promotes a cellular environment which results in reduced mitochondrial oxidants and preserves mitochondrial function. Following DOX treatment, the mitochondrial function readout of the carvedilol pre-treated cells was similar to the readout of cells that overexpressed the mitochondrial biogenesis regulator PGC-1α. We elucidated an unrecognized redox-regulatory property of carvedilol that can contribute to cytoprotection and the preservation of respiratory capacity that would otherwise diminish following DOX treatment.

## 2. Materials and Methods

### 2.1. Chemicals and Reagents

Dulbecco’s modified Eagle’s medium (DMEM), paraformaldehyde, DOX, gelatin and Hoechst 33342 were purchased from Sigma-Aldrich (St. Louis, MO, USA). mTeSR1™ media, CHIR99021 and IWP-2 were purchased from StemCell Technologies (Vancouver, BC, Canada). Matrigel matrix was purchased from Corning Inc. (Corning, NY, USA). Carvedilol and ROCK inhibitors were purchased from Selleck Chemicals (Houston, TX, USA). CytoTune™-iPS 2.0 Sendai Reprogramming Kit, Fetal Bovine Serum (FBS), AlamarBlue™ cell viability reagent, serum-free B-27 supplement, B-27 supplement minus insulin, TrypLE™ express enzyme and RPMI 1640 [+] L-Glutamine medium were purchased from ThermoFisher Scientific (Waltham, MA, USA).

### 2.2. Cell Culture

Commercially available rat cardiac myoblast (H9c2) cells were purchased from American Type Culture Collection (ATCC; Rockville, MD, USA). H9c2 cells were cultured in Dulbecco’s Modified Eagle Medium (DMEM) with 10% fetal bovine serum (FBS), 100 units penicillin/mL and 100 µg streptomycin/mL (P/S) at 37 °C in a humidified atmosphere with 5% CO_2_, and the medium was changed every 2–3 days. The cells were trypsinized and plated (25,000–30,000 cells/cm^2^) in a 96-wells plate for cell viability and in a 12-wells plate for Western blot analyses.

### 2.3. Human iPSCs

#### 2.3.1. Reprogramming Human Fibroblasts to iPSCs

All studies using human and genetically modified cells in the laboratory were performed with appropriate institutional review board (IRB) approval at the Medical College of Wisconsin (Milwaukee, WI, USA). For this study, informed consent was obtained from all subjects for the generation human iPSC-derived cell lines as described in our previous publication [36]. Dermal fibroblasts were isolated from a healthy male adult and expanded in DMEM supplemented with 10% FBS and P/S (all from Life Technologies, Carlsbad, CA, USA). Standard reprogramming into iPSCs was performed with CytoTune™-iPS 2.0 Sendai Reprogramming Kit for the transduction of the genes encoding KOS, hc-Myc, and hKLF4 [37]. Transduced fibroblasts were next seeded onto clonally derived SNL feeder cells (Cell Biolabs, San Diego, CA, USA) that were mitotically inactivated through treatment with Mitomycin C (ATCC) in an embryonic stem cell (ESC) medium composed of DMEM supplemented with 20% KnockOut™ serum replacement, minimum essential medium (MEM) non-essential amino acids, 2 mM L-glutamine (all from Life Technologies), P/S, 0.1 mM β-mercaptoethanol (Sigma), 10 ng/mL human basic fibroblast growth factor (FGF) (Cell Signaling Technology, Danvers, MA, USA) and 50 ng/mL L-ascorbic acid (Sigma). The resulting human iPSC clones were manually picked and maintained on feeder cells in the ESC medium for four passages before transitioning to feeder-free culture, as described below.

#### 2.3.2. iPSC Culture

iPSCs were routinely cultured under feeder-free conditions on Matrigel (Corning, Corning, NY, USA)-coated dishes with serum-free mTeSR1 (Stem Cell Technologies, Vancouver, BC, Canada). Cells were passaged every 3–4 days using TrypLE (Life Technologies) and seeded in the medium containing 10 μM Rho-associated kinase inhibitor (Y-27632, Selleck) for 24 h following passage.

#### 2.3.3. iPSC-CM Differentiation

A previously described protocol using chemical modulation of the Wnt pathway was performed for iPSC-CM differentiation [38]. Briefly, iPSCs at 100% confluency were overlaid with either Matrigel in mTeSR1 or StemMACS iPS-Brew XF medium on day minus 1. On day 0, cells were treated with 7 μM Chir-99021 (Selleck, cat#S2924) in RPMI minus insulin medium (RPMI supplemented with 1× B27 supplement minus insulin; both from Life Technologies) to activate the canonical Wnt signaling pathway. On day 2, the medium was changed to RPMI minus insulin. On day 3, cells were treated with 5 μM of the Wnt signaling inhibitor IWP-2 (Stem Cell Technologies, cat#72122) in RPMI minus insulin, with the medium subsequently changed on day 5 to RPMI minus insulin. The medium was changed to the complete medium, and RPMI was supplemented with 1× B27 supplement (Life Technologies, cat#17504044) on day 7 and repeated every 2–3 days thereafter. Cells were typically utilized for experiments ~30 days post differentiation. Human iPSC-CMs were passaged using TrypLE Express (Life Technologies, cat#12605010) and seeded for experiments in a complete medium containing 5% FBS and 5 μM ROCK inhibitor on 0.1% gelatin-coated surfaces.

### 2.4. Determination of Cell Viability

Four experimental groups were studied at standard culture conditions as follows: (1) untreated control cells; (2) pre-treatment of cells with carvedilol for 3 h with variable concentrations before doxorubicin was added for 48 h; (3) cells were treated with carvedilol alone for a total of 48 h; and (4) cells were treated with doxorubicin alone for 48 h. Cell viability was evaluated by the AlamarFijiBlue assay.

### 2.5. Caspase 3/7 Assay

H9c2 cells were seeded on coverslips and cultured in a 24-wells plate. Cells were pretreated with 1 µM carvedilol for 3 h before the DOX treatment for 24 h. To assess viability, cells were incubated in 2 μM CellEvent Caspase-3/7 Green Detection Reagent (Invitrogen, Waltham, MA, USA, cat#R37111) in PBS for 30 min before they were counterstained with Hoechst 33342 (1:500) (ThermoFisher Scientific, cat#62249) for 10 min at 37 °C. After staining, cells were preserved with 4% paraformaldehyde for 15 min. The fluorescent signals were observed by the fluorescence microscope (Nikon, Tokyo, Japan). The total cell number was obtained by counting stained nuclei. The caspase 3/7 positive cell ratio was generated as the number of caspase 3/7-positive cells/total number of cells.

### 2.6. Detection of Mitochondrial Oxidants

Following the treatment with either 0.5 µM DOX and/or 1 µM carvedilol, cells were incubated with a 5 µM MitoSOX reagent (ThermoFisher, cat#M36008) for 10 min at 37 °C, washed three times with warm Hanks’ balanced salt solution/Ca^2+^/Mg^2+^ buffer (ThermoFisher, cat#14025092) and incubated with Hoechst 33342 (1:500) for another 10 min at 37 °C to label nuclei. Cells were then washed three times and fixed with a 4% paraformaldehyde. The cells were washed twice with Hanks’ balanced salt solution and for all analyses, red fluorescence (MitoSOX) and blue fluorescence (Hoechst) were captured using an Olympus microscope. Intracellular quantitative analysis was performed using Fiji ImageJ software (version 2.9.0; 14 September 2022). For comparison between treatments, the average mean measurement of the red fluorescence intensity of MitoSOX fluorescence was normalized to the cell number by counting the number of nuclei per field.

### 2.7. Immunocytochemistry

Cells on glass coverslips were fixed with 4% paraformaldehyde (Sigma) for 20 min at 37 °C, permeabilized with 0.3% Triton X-100 (Sigma, cat#T8532), blocked with 3% bovine serum albumin (BSA; ThermoFisher, cat#15260037), and stained appropriately before finally being mounted with the Antifade mounting medium plus DAPI (Vectashield, Burlingame, CA, USA, cat#H1200). The cells were stained with the following primary antibodies overnight at 4 °C: Nrf2 (Abcam, ab137550) (1:100); Keap1 (ThermoFisher, MA5-17106) (1:100). After several washes in 0.3% triton in PBS, the cells were incubated in the dark for one hour at room temperature with secondary antibody. Secondary antibodies were as follows: Alexa Fluor 488 donkey anti-rabbit IgG and Alexa Fluor 555 donkey anti-mouse IgG (Life Technologies, 1:500). After several washes, cells were mounted before imaging. Cardiac troponin T (cTNT; Invitrogen, cat#MA5-12960) (1:100) was used to stain iPSC-CMs. Images were taken on a Nikon 55i fluorescence microscope.

### 2.8. Western Blot Analysis

After treatment, cells were lysed using a RIPA lysis buffer (ThermoFisher) containing Halt protease and phosphatase inhibitor cocktail (ThermoFisher). Equal amounts of total protein were separated on Mini-Protean TGX gels, 4–20% (Bio-Rad) and transferred to PVDF membranes. Membranes were blocked with nonfat dry milk and probed with the following primary antibodies: Keap1 (Cell Signaling # 7705S, 1:500); PGC-1α (Abcam, Cambridge, UK, ab106814, 1:500); GAPDH (Cell Signaling Technology, 2118, 1:10,000), followed by incubation with HRP-conjugated secondary antibody. Protein bands were detected by ECL chemiluminescence. Quantification of band intensity was performed using ImageJ software.

### 2.9. Quantitative Polymerase Chain Reaction (qPCR)

Cells were lysed directly in the culture dish by the addition of TRIzol reagent (ThermoFisher Scientific) and RNA was extracted according to the manufacturer’s instructions. Harvested RNA was subsequently ethanol precipitated as described previously [39]. The concentration of extracted total RNA was quantified and qualified by a NanoDrop 2000 spectrophotometer and reverse transcribed into cDNA using the iScript cDNA Synthesis kit (BIO-RAD). qPCR was carried out using PowerUp SYBR Master Mix and QuantStudio 6 (ThermoFisher Scientific) to assess relative gene expression. The relative amounts of mRNA were determined based on 2^−∆∆Ct^ calculations with GAPDH as the endogenous reference. All samples were run in quadruplicate and the melting point dissociation curves were generated to confirm the specificity of the amplified product (Table 1).

### 2.10. Measurement of Mitochondrial Oxygen Consumption Rate

To investigate the bioenergetic consequences of the treatments tested, the oxygen consumption rate (OCR; reported in the unit of picomoles O_2_ per minute) was measured in real-time using a Seahorse XFe96 Analyzer per the manufacturer’s protocols. iPSC-CMs were seeded into Seahorse XF-96 plates (Agilent Technologies, Santa Clara, CA, USA) coated with 0.1% gelatin at a density of 3.5 × 10^4^ cells/well on day 1. Fresh media were added to wells on day 3 without removing spent media and on day 5, spent media were replaced with 90 μL of media that contained either DMSO or 2.5 µM carvedilol. DOX (10 µL of 5 µM) was added to the respective wells four hours later to yield a final DOX concentration of 0.5 μM and the total medium volume of 100 μL. A seahorse assay was performed the following day. On the day of the assay, the OCR values were measured in Seahorse XF DMEM (180 µL/well) containing: 25 mM glucose, 2 mM glutamine and 1 mM pyruvate, pH 7.4. After incubating in a CO_2_-free incubator (37 °C) for 30–45 min, respiration measurements were started and cells were exposed to the sequential addition of: 20 µL of 15 µg/mL (19 µM) oligomycin, 22.2 µL of 10 µM FCCP and 24.6 µL of 10 µM rotenone + 10 µM antimycin A (final concentrations: 1.9 µM oligomycin, 1 µM FCCP and 1 µM rotenone plus 1 µM antimycin A). After the completion of OCR measurements, Hoechst staining was given as a final injection for image-based cell counting. Mitochondrial respiratory parameters were analyzed using the Seahorse Wave software (Agilent Technologies, Santa Clara, CA, USA; Version 2.6.1). The OCR values were normalized to cell count in the corresponding wells, determined by fluorescence imaging of the plate and spot counting using Cytation 1 instrument (BioTek, Winooski, VT, USA).

### 2.11. Measurement of Intracellular Reduced Thiols

To measure the total level of intracellular reduced thiols, we used monochlorobimane reagent (MCB) (Sigma-Aldrich) which is nonfluorescent until conjugated to thiols. Cells were incubated with 50 µM MCB for 40 min. The fluorescence intensity of conjugated MCB was measured using a fluorescence plate reader (exc. 398 nm; emi. 488 nm).

### 2.12. Statistical Analysis

Each experiment was typically repeated several times, with technical replicates completed in triplicate or greater. Data were presented as the mean ± SD of the technical replicates and representative of the indicated number of independent experiments, n, unless otherwise noted. Statistical comparisons were made by Student’s *t*-test for two different groups or one-way analysis of variance (ANOVA) for multiple group comparisons, as appropriate, with GraphPad Prism 9 software. Tukey’s or Dunnett’s multiple comparisons test was used when appropriate to determine whether there was a difference between the mean of all possible pairs. The differences were assumed statistically significant when *p* ≤ 0.05.

## 3. Results

### 3.1. Carvedilol Exerts Cardioprotective Effects from CTRTOX in Rat Cardiomyoblasts and Human iPSC-CMs

#### 3.1.1. Cell Viability Assay

**Figure 1:**
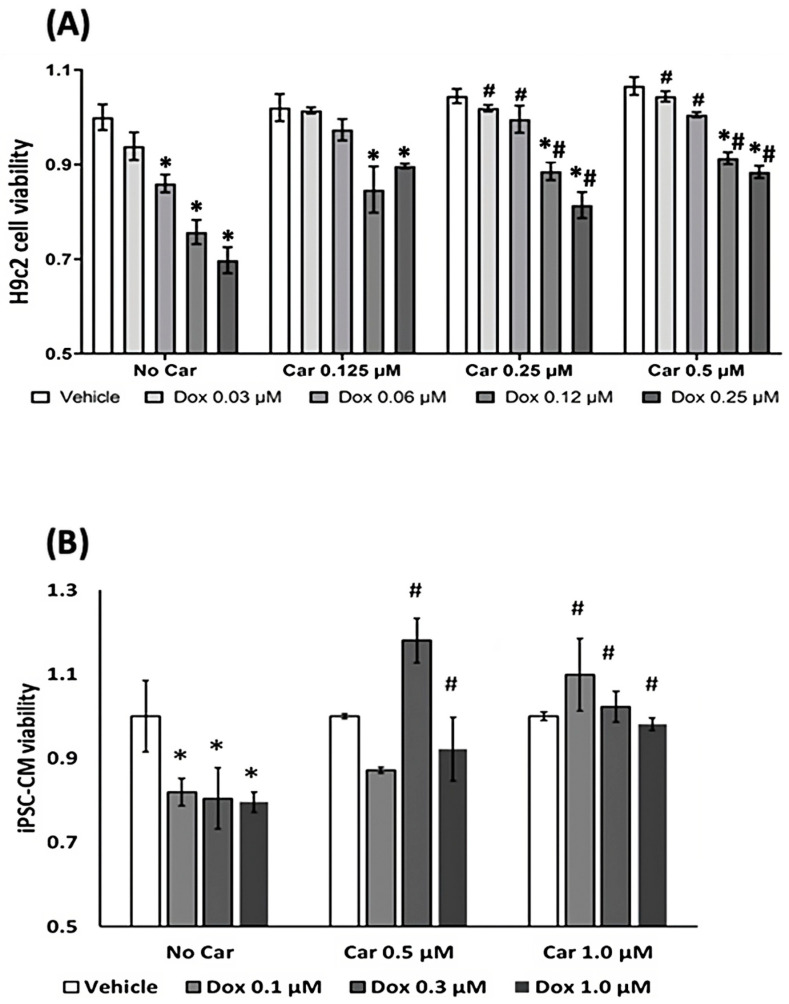
Determination of dose-dependent carvedilol on cell viability for prevention of DOX-induced cell death in (**A**) H9c2 cells and (**B**) human iPSC-CMs. Cells were pretreated with carvedilol for 3 h followed by DOX for 48 h prior to Alamar blue assay. Data are normalized to average value of vehicle and presented as mean ± SEM. Data are representative of three independent experiments, with each treatment group completed in triplicate. (ANOVA; * *p* ≤ 0.05 vs. vehicle group; # *p* ≤ 0.05 vs. no carvedilol group).

DOX-induced cytotoxicity can be reflected in the measurement of the resultant cell viability. We propose that carvedilol pretreatment would attenuate DOX toxicity, conferring cytoprotection. To assess cell protection, we first incubated H9c2 cells and human iPSC-CMs for 3 h with carvedilol followed by 48 h DOX treatment before performing an assessment of the cell viability using the AlamarBlue assay (Figure 1). Carvedilol treatment alone had no effect on cell viability. The cardioprotective effects of carvedilol against CTRTOX were dose-dependent in both cell lines with iPSC-CMs attaining complete protection from DOX-induced loss of cell viability at 1 µM concentration. The cardioprotective effects were observed under all conditions, reaching statistical significance at most concentrations. Whereas the cell viability of H9c2 cells was stable over the pharmacologic range for carvedilol, higher doses of carvedilol led to a decrease in cell viability with an EC_50_ > 10 µM after 72 h treatment (Figure S2B). Accordingly, all subsequent experiments were performed at the dynamic ranges between 0.25 and 2.5 µM carvedilol to assess the pharmacological effects on cell viability for H9c2 cells (Figure 1A) and human iPSC-CMs (Figure 1B), respectively.

#### 3.1.2. Apoptosis Assay

**Figure 2:**
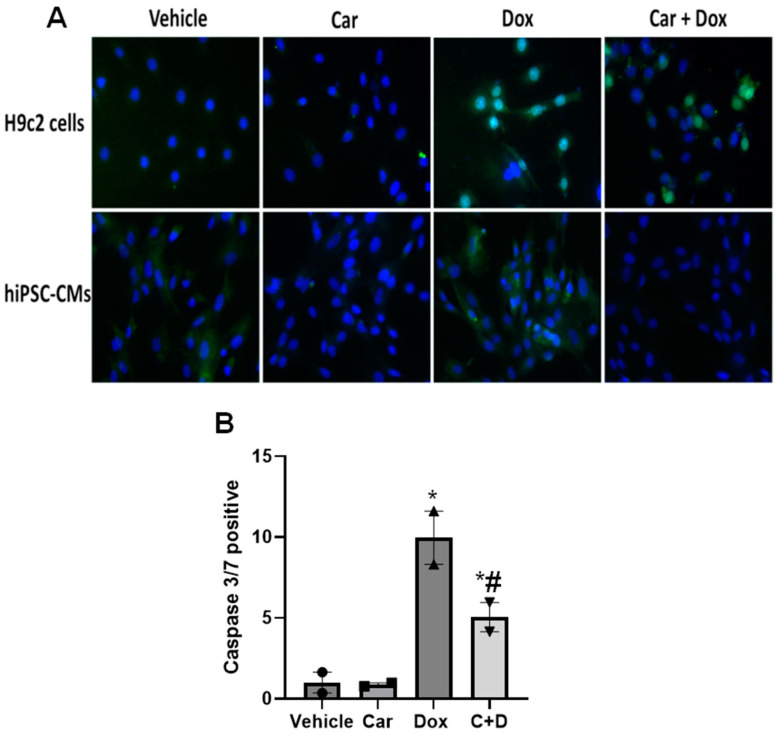
Determination of carvedilol for prevention of DOX-induced apoptosis using caspase 3/7 activity in (**A**) H9c2 cells and human iPSC-CMs with (**B**) quantification of caspase 3/7 positive H9c2 cells. Cells were pretreated with 1 µM carvedilol for 3 h followed by 24 h with 0.5 µM DOX before the CellEvent Caspase 3/7 assay. Scale bar = 50 µm. Quantification data are presented as mean ± SEM of two independent experiments, with each treatment group completed in triplicate. (ANOVA; * *p* ≤ 0.05 vs. vehicle; # *p* ≤ 0.05 vs. DOX).

DOX can reduce cardiomyocyte viability and induce apoptosis. To evaluate the prevention of apoptosis, cells were incubated for 3 h with 1 µM carvedilol followed by 24 h of 0.5 µM DOX treatment before giving the CellEvent caspase-3/7 detection reagent, which permeates the cell and labels apoptotic cells with a bright, fluorescent signal. The cells in green were positive for the apoptosis marker while DAPI was used to stain the nuclei blue (Figure 2). The total number of cells, both labeled with the apoptosis marker and unlabeled, were quantified and ratios were generated as the number of apoptosis-positive stains and the total number of cells (DAPI stain). We found that carvedilol pretreatment reversed the number of caspase 3/7 positive cells induced by 24 h DOX exposure both in H9c2 cells and in human iPSC-CMs (Figure 2). The number of caspase 3/7 positive H9c2 cells significantly decreased by about half with carvedilol pretreatment before DOX compared to DOX exposure alone (Figure 2B). These results suggest that carvedilol pretreatment reduces DOX-induced toxicity, preventing cell death via apoptosis.

### 3.2. Carvedilol Pretreatment Decreases Levels of Mitochondrial Oxidants following DOX Exposure in H9c2 and Human iPSC-CMs

**Figure 3:**
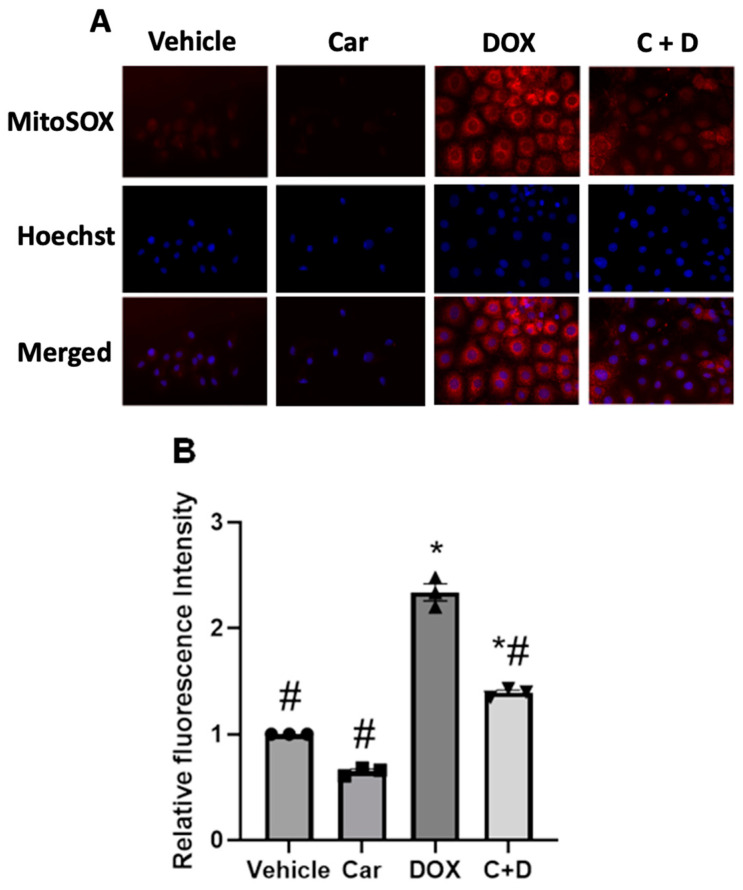
Effect of carvedilol on DOX-induced mitochondrial oxidants in H9c2 cells. Cells were treated for 24 h with 1 µM DOX with or without 3 h pretreatment with 1 µM carvedilol. Red fluorescence represents the extent of MitoSOX Red oxidation. (**A**) Representative images and (**B**) quantification of MitoSOX intensity in cytoplasmic space (scale bar = 50 µm). The intensity data are presented as mean ± SEM (n = 3). (ANOVA; # *p* ≤ 0.05 vs. DOX; * *p* ≤ 0.05 vs. vehicle).

Both improvements in cell viability and prevention of cell death by carvedilol from doxorubicin treatment could be related to alterations of redox balance and proteostatic imbalances of mitochondrial proteins, resulting in impaired mitochondrial function [40]. To first determine the redox effects of DOX treatment, we evaluated MitoSOX Red oxidation as a biosensor of mitochondrial oxidants following 1 µM DOX treatment for 24 h either with or without 3 h pretreatment with 1 µM carvedilol. MitoSOX staining in DOX-treated H9c2 cells resulted in increased MitoSOX red fluorescence staining, indicating a shift towards a more oxidizing environment (Figure 3A). The increased (~2.5-fold) MitoSOX-derived red fluorescence signal observed after DOX treatment was significantly decreased by cell pretreatment with carvedilol (Figure 3B; # *p* < 0.05 and * *p* < 0.05, respectively). Likewise, the MitoSOX red fluorescence staining in iPSC-CMs qualitatively exhibited an increase following DOX treatment and a reversal with prophylactic carvedilol, respectively (Figure S5), supporting the differential levels of mitochondrial oxidants. These results indicate that potent changes of the intracellular redox poise are elicited during both DOX and carvedilol treatments, which we hypothesize to be dynamically linked to the redox-sensitive Nrf2/Keap1 regulatory pathways.

### 3.3. Carvedilol Pretreatment Sustains Levels of Cytosolic Keap1 and Decreased Nuclear Translocation of Nrf2 in DOX-Treated Rat Cardiomyoblasts and Human iPSC-CMs

**Figure 4:**
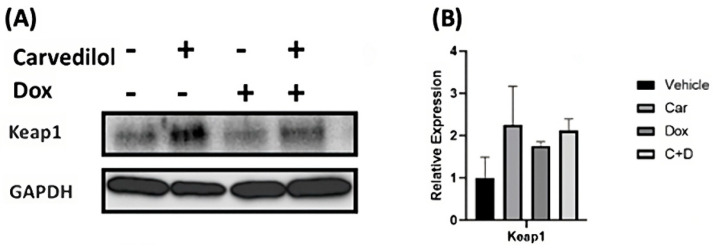

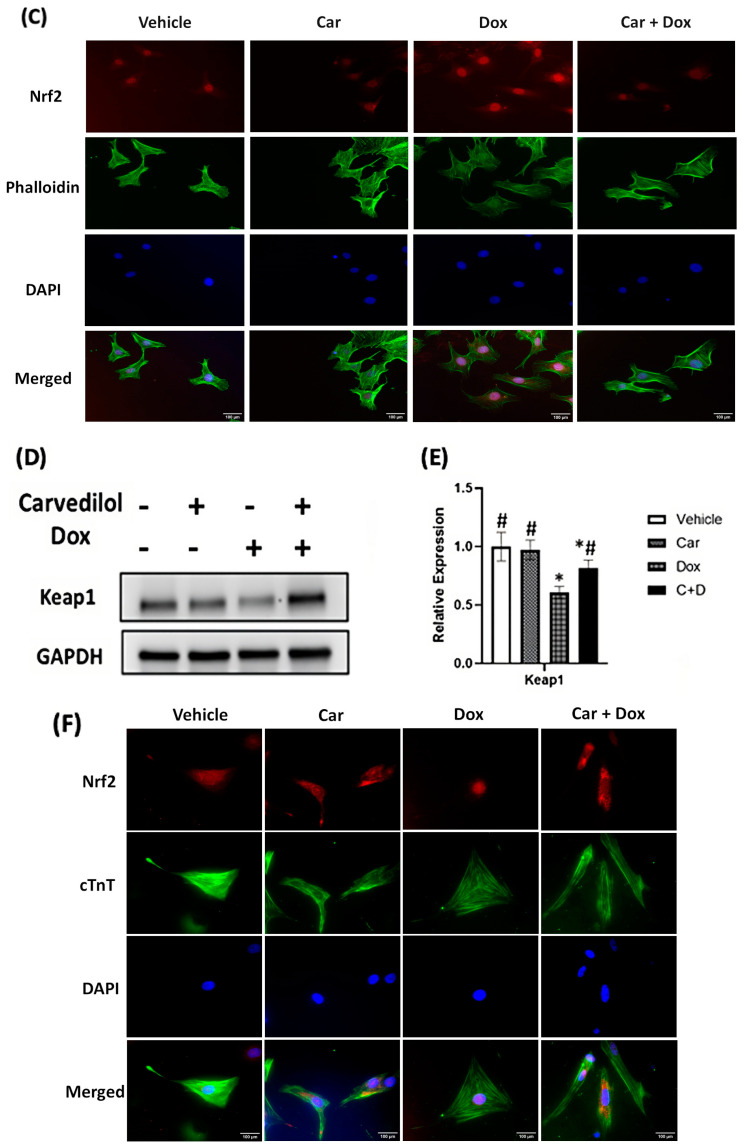
Effect of differential treatment on the Nrf2/Keap1 system in H9c2 cells and human iPSC-CMs. Cells were pretreated with 1 µM carvedilol for 3 h before 0.5 µM DOX for 6 h. (**A**) Representative images and (**B**) quantitative analysis of Keap1 protein expression levels in H9c2 cells. (**C**) Localization of Nrf2 (red) and Keap1 (green) in H9c2 cells. (**D**) Representative images and (**E**) quantitative analysis of Keap1 protein expression levels in human iPSC-CMs. (**F**) Localization of Nrf2 (red) in human iPSC-CMs. The magnification of the immunocytochemistry images was 40×. Scale bar = 50 μm. cTnT (green; in (**F**) only) was used as a cardiomyocyte marker. DAPI (blue) was used to stain nuclei. Quantification data are presented as relative expression levels after normalization to GAPDH and as means ± SEM from at least three independent experiments. (ANOVA; * *p* ≤ 0.05 vs. vehicle. # *p* ≤ 0.05 vs. DOX).

The well-established NFE2L2/KEAP1 system has emerged as the cellular stress-sensitive master transcriptional regulator of various downstream targets, predominantly genes encoding anti-oxidative and detoxification pathways, associated with both cancer and cardiovascular susceptibility in humans [41,42]. To assess the effects of pharmacological treatments on subcellular localization, immunocytochemical studies revealed the translocation of Nrf2 into the nucleus (i.e., an increased red intensity) after DOX treatment alone, which is readily reversed by carvedilol pretreatment (Figure 4C,F). Because these cellular changes were only observed with pretreatment but not simultaneous cotreatment, such effects of carvedilol implicate potential post-translational events for the prevention of DOX-dependent nuclear localization of Nrf2 in H9c2 cells (Figure S4).

### 3.4. DOX Treatment Induces Expression of Nrf2-Regulated Genes in Human iPSC-CMs

**Figure 5:**
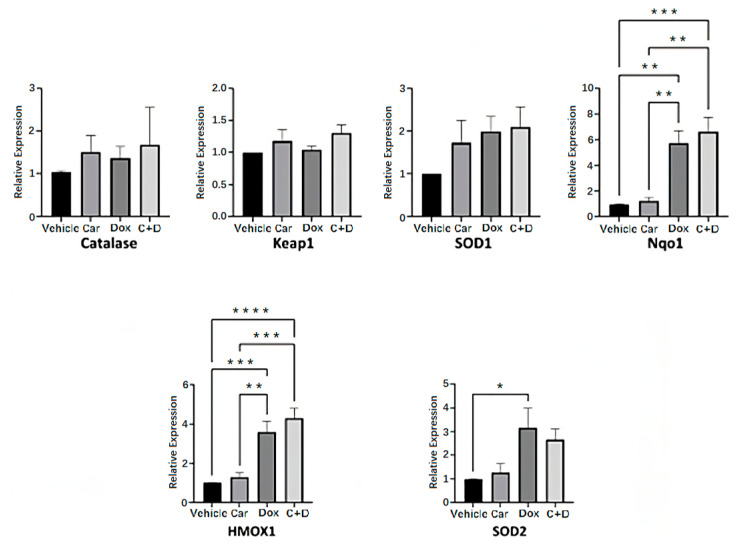
Gene expression analysis of Nrf2-associated antioxidant genes following differential treatment in human iPSC-CMs. iPSC-CMs were pretreated with 2.5 µM carvedilol for 4 h before 24 h treatment with 0.5 µM DOX. Data are normalized to housekeeping gene GAPDH and vehicle group and presented as mean ± SEM from at least three independent experiments. (ANOVA; * *p* ≤ 0.05, ** *p* ≤ 0.01, *** *p* ≤ 0.001, **** *p* ≤ 0.0001).

In response to either oxidative and/or electrophilic stresses, Nrf2 translocates to the nucleus where it binds to the antioxidant response element (ARE) to regulate the expression of a plethora of antioxidant response and cytoprotective genes. The Nrf2-regulated antioxidant response is largely appreciated for its subcellular distribution rather than its induction [43,44] and since we found DOX treatment results in the nuclear localization of Nrf2, we sought to assess the expression of Nrf2-regulated genes as an indirect assessment of Nrf2 activation. Human iPSC-CMs were pre-treated for 4 h with either 2.5 µM carvedilol or vehicle followed by 24 h treatment with 0.5 µM DOX before harvesting of nucleic acids for gene expression analysis. We found that 0.5 µM DOX treatment resulted in the increased expression of several (e.g., Nqo1, HMOX1, SOD2) but not all Nrf2-regulated genes (Figure 5). This is consistent with reports that Nrf2 activation does not necessarily alter the expression of all its target genes. There exists a complex network of other transcriptional factors whose cellular signaling may regulate the activation of different subsets of genes [45]. While we propose that carvedilol pretreatment reduces cellular oxidants and nuclear Nrf2 localization, our gene expression analysis reveals that DOX treatment results in the activation of the antioxidant response, whether or not there was prior treatment with carvedilol. It is possible that while carvedilol exerts protective effects, activation of the antioxidant response may still be warranted for cell survival so long as there is the presence of DOX.

### 3.5. Carvedilol Pretreatment Prevents DOX-Induced Decreased Levels of Total Reduced Thiols in H9c2 Cells

Thiols are important functional groups used not only by many cellular components including antioxidant enzymes, but they maintain the highly reducing environment of the cytosol under physiological conditions. To assess the effects on intracellular reduced thiols, we employed the monochlorobimane reagent that readily reacts with low molecular weight thiols, including the major and most abundant glutathione. We pretreated H9c2 with variable carvedilol doses for 2 h followed by 4 h incubation with 0.5 µM DOX. Though carvedilol treatment alone had no effects on thiol homeostasis under non-stress conditions, we found that carvedilol not only prevented the expected DOX-induced decreases in total intracellular thiols but the effects on thiol imbalances were fully reversed in a dose-dependent manner (Figure S6).

### 3.6. Carvedilol Prevents DOX-Induced Suppression of Mitochondrial Respiratory Function in Human iPSC-CMs

**Figure 6:**
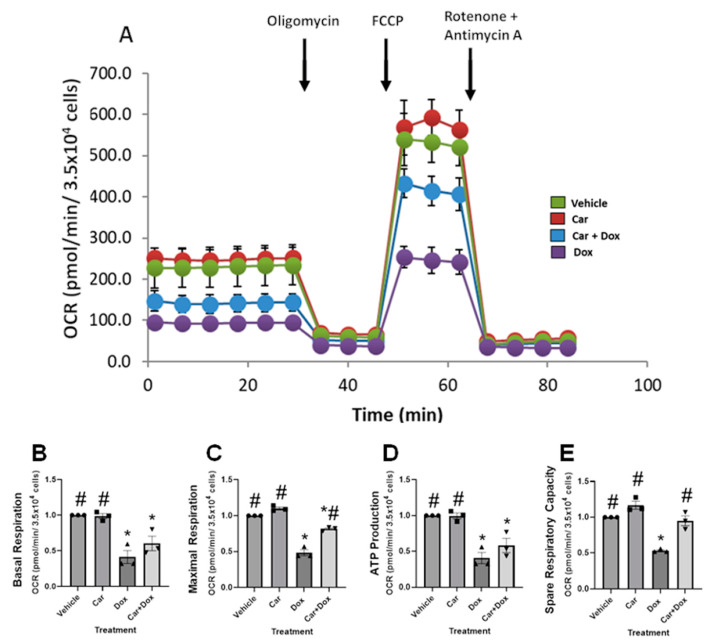
The effect of carvedilol, DOX and/or both on the mitochondrial respiration of human iPSC-CMs. iPSC-CMs were pretreated for 4 h with either DMSO or 2.5 µM carvedilol before 20 h treatment with 0.5 µM DOX. The oxygen consumption of mitochondrial respiration was measured as pmol O_2_/min/(3.5 × 10^4^ cells). (**A**) Representative oxygen consumption rate profile in intact iPSC-CMs after 24 h total treatment. Arrows indicate the sequential addition of oligomycin, FCCP and rotenone plus antimycin A. Graph showing key parameters of mitochondrial function including (**B**) basal respiration, (**C**) maximal respiration, (**D**) ATP production-linked respiration and (**E**) spare respiratory capacity all expressed as pmol O_2_/min. (ANOVA; * *p* ≤ 0.05, versus vehicle group. # *p* ≤ 0.05, versus DOX group). Data in graphs are normalized to vehicle and presented as mean ± SEM of three independent experiments, with each treatment group completed in quadruplicate. Car, carvedilol; Dox, doxorubicin; OCR, oxygen consumption rate; FCCP, carbonyl cyanide-4-(trifluoromethoxy)phenylhydrazone.

Because cancer therapy-induced cardiovascular complications such as heart failure are often late onset and with lethal outcomes, prevention strategies are actively being pursued to improve optimal outcomes, especially if detected and implemented early [46]. How antineoplastics such as doxorubicin, during cancer treatment, alter tissue metabolism and/or contribute to imbalances of energetic networks has not hitherto been addressed at the cellular levels. To determine the effects on mitochondrial respiration, we tested the hypothesis that carvedilol pretreatment would not only mitigate DOX-induced decreases in the mitochondrial oxygen consumption rate (OCR) but the metabolic perturbations are both necessary and sufficient to reverse chemotherapy-induced toxicity. Cells were pretreated with either 2.5 µM carvedilol or DMSO for 4 h before overnight DOX (0.5 µM) treatment for 20 h for measurements of the oxygen consumption rate (OCR) as an indicator of mitochondrial respiration using a Seahorse XFe96 analyzer (Billerica, MA, USA). Oxygen consumption is a parameter of mitochondrial bioenergetics and all drugs were washed out immediately before the Seahorse assay (Figure 6A). Though 2.5 µM carvedilol treatment by itself had no impact on basal respiration, 0.5 µM DOX treatment significantly decreased mitochondrial function compared with vehicle treatment (Figure 6). When the suppression of maximal respiration and ability to produce ATP by DOX treatment were evaluated after the pretreatment with carvedilol, these preventative effects were partial and/or significantly improved for basal respiration (Figure 6B), maximal respiration (Figure 6C), ATP production (Figure 6D) and spare respiratory capacity (Figure 6E).

### 3.7. PGC-1α Overexpression Recapitulates the Pharmacological Prevention by Carvedilol of DOX-Induced Mitochondrial Dysfunction in Human iPSC-CMs

**Figure 7:**
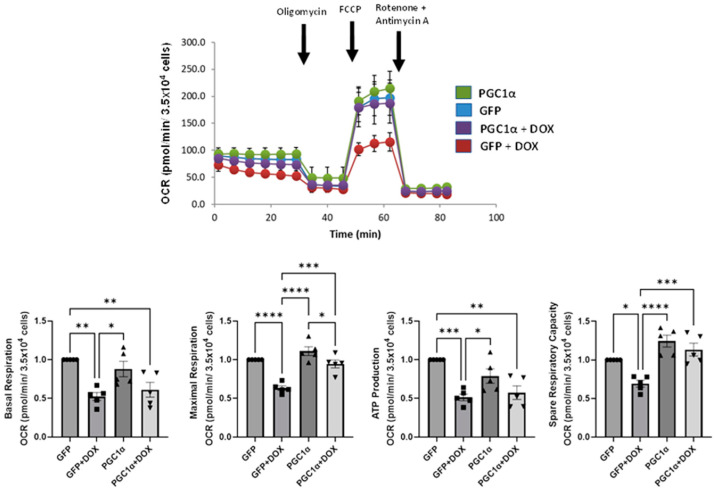
OCR profile in lentiviral transduction of PGC-1α in human iPSC-CMs. Arrows indicate the sequential addition of oligomycin, FCCP and rotenone plus antimycin A. OCR profile plot is representative of five independent experiments and data in graphs were normalized to GFP control and presented as mean ± SEM. (ANOVA; * *p* ≤ 0.05, ** *p* ≤ 0.01, *** *p* ≤ 0.001, **** *p* ≤ 0.0001) OCR, oxygen consumption rate; FCCP, carbonyl cyanide-4-(trifluoromethoxy)phenylhydrazone.

Since the foregoing evidence indicates carvedilol pretreatment and mitigates the deleterious effects of DOX by directly enhancing DOX-induced suppression of mitochondrial respiratory, we next tested the hypothesis that such pharmacological effects on mitochondrial OCR will be recapitulated by genetic manipulations of the transcriptional coactivator PGC-1α, the master regulator of mitochondrial biogenesis, in human iPSC-CMs. Indeed, carvedilol’s amelioration of mitochondrial dysfunction was partially recapitulated by PGC-1α overexpression (Figure 7). While PGC-1α overexpression did not prevent the DOX-induced decrease in basal and ATP-linked respiration, similar to carvedilol, it did restore maximal respiratory capacity in iPSC-CMs. These results demonstrated that effects of DOX-induced mitochondrial dysfunction could be attenuated by carvedilol pretreatment and genetic maneuvers, aimed at PGC-1α induction. Several attempts to assess the levels of PGC-1a by immunoblotting were equivocal and inconclusive under our experimental conditions.

## 4. Discussion

The present study has provided direct evidence that carvedilol pretreatment not only attenuates DOX-induced cytotoxicity but addresses the cellular mechanisms underlying mitochondrial respiratory function and metabolism using human iPSC-CMs in vitro. Although recognized for both antioxidant and ROS-scavenging properties [34,47], how carvedilol has garnered attention for cardioprotection remains elusive. We believe that our report of carvedilol’s ability to achieve cytoprotection, maintain redox homeostasis, alter cellular mitochondrial respiration and, for the first time, to recapitulate the mitochondrial-protective effects that are achieved by the activation of the master regulator, PGC-1α, for the preservation of mitochondrial function during DOX in vitro heralds novel opportunities for drug development and translation into the clinic.

Because cardiotoxicity is the second leading cause of mortality and morbidity for cancer survivors [48], prophylactic maneuvers to prevent cardiotoxicity and related adverse side effects during cancer treatment have had a storied history in cancer and cardiovascular (i.e., termed cardio-oncology) fields. Decades earlier, multiple prospective randomized clinical trials of patients with metastatic breast cancer with a prior cumulative dosage of DOX 300 mg/m^2^ provided the FDA evidence of clinical efficacy to approve dexrazoxane, an intracellular chelating agent, for the prevention of life-threatening cardiomyopathy during cancer therapy [49,50]. However, the lack of efficacy in children and concerns raised about reducing the tumor-killing capacity of DOX [4] have restricted dexrazoxane’s use primarily for adult patients receiving cumulative anthracycline therapy with either advanced or metastatic cancer [21].

Accordingly, safe and effective cardioprotective drugs for prophylaxis have considerable clinical appeal and are urgently needed to reduce CTRTOX. β-blockers are established treatments for the guideline-based medical management and treatment of heart failure with left ventricular dysfunction (LV < 40%) including DOX-induced cardiotoxicity. As multiple studies including clinical trials have focused on the prevention of chemotherapy-induced cardiomyopathy, carvedilol has a widespread appeal not only for the potential properties to prevent DOX-induced myocardial injury but to prevent LV dysfunction accompanying cancer treatment. In the recent Carvedilol for the Prevention of Chemotherapy-related Cardiotoxicity: the CECCY Trial, patients (n = 200) with HER2-negative breast cancer and normal left ventricular ejection fraction (LVEF), who were prospectively randomized and double-blinded to receive either carvedilol or placebo until chemotherapy completion, developed the primary endpoint at similar rates (13.5% vs. 14.5% incidence of prevention of >10% LVEF at 6 months) but had significantly reduced amounts for the secondary endpoints such as troponin I and diastolic dysfunction [33]. While dose escalation of carvedilol might have been poorly tolerated (e.g., blood pressure, heart rate) in the CECCY Trial [51], our studies have investigated the drug’s salutary effects for modifying the cellular redox status, maintaining ‘redox poise’ and mitochondrial metabolism by using human iPSC-derived cardiomyocytes for greater translational relevance and disease modeling of chemoprevention by DOX-induced oxidative stress while contributing substantially to cardioprotection.

Herein, we have shown that carvedilol pretreatment expectedly prevents DOX-induced apoptosis in both H9c2 cells and human iPSC-CMs in a dose-dependent manner (Figure 1). Such pretreatment also counteracts the detrimental effects of ROS such as proteotoxicity and DNA damage. It also preserves cell viability, ATP metabolic functions and prevents DOX-induced mitochondrial oxidants and dysfunction (Figure 3 and Figure 4) [27,52,53,54].

The nuclear factor erythroid-derived 2-like 2-Kelch-like ECH-associated 1 (Nrf2-Keap1) system has major roles at the intersections not only for cancer biology as a molecular target for certain tumors (i.e., termed synthetic lethality), but also for the antioxidant and antiapoptotic pathways used for cardioprotection [55,56,57]. Nrf2 is a major transcriptional regulator of multiple cytoprotective genes encoding antioxidant enzymes, metabolic enzymes, drug transporters, detoxification enzymes and inflammatory pathways. As a cysteine thiol-rich protein, Keap1 is a redox-sensitive, negative regulator of Nrf2 by promoting its ubiquitination via the adaptor of the Cullin 3 (Cul3-)-ubiquitin E3 ligase [58]. Under basal conditions, low levels of Nrf2 expression and short half-life (<20 min) are physiologically maintained in both organs and tissues through the rapid degradation of ubiquitinated Nrf2 by the 26S proteasome [58,59]. Toxic chemicals and electrophiles promote the shift toward oxidative stress and trigger rapid translocation of Nrf2 into the nucleus where it binds to antioxidant response elements (AREs) of target genes including NAD(P)H dehydrogenase [quinone] 1 (*Nqo1*), heme oxygenase 1 (*HMOX1*) and superoxide dismutase 1, -2 (*SOD1*, *-2*) (Figure 5) [55]. The targeted upregulation of gene expression of this endogenous antioxidant response results in potent antioxidant and cytoprotective activities to effectively ameliorate DOX-induced oxidative stress, which is similarly used by potent Nrf2 inducers such as resveratrol [60,61], sulforaphane [62,63,64] and bardoxolone-methyl [65,66]. Our study is the first to report decreased Keap1 protein expression following acute DOX treatment in human iPSC-CMs, which is consistent with newly synthesized Nrf2 escaping Keap1-dependent ubiquitination and rapid proteasomal degradation (Figure 4). Our gene expression analysis revealed no significant effects of DOX or carvedilol treatment on levels of Keap1 mRNA. Therefore, the plausible mechanisms for the protective efforts are carvedilol’s ability to recruit the redox-dependent interactions between Keap1 and Nrf2, thereby ensuring the maintained redox homeostasis during oxido-reductive perturbations (Figure 5). Despite its apparent and well-known antioxidant properties, our results suggest that carvedilol does not reverse Nrf2 activation, thereby maintaining cellular stress response activity at high levels. We posit that the protective effects of carvedilol are primarily to preserve bioenergetic function and cell viability, which collectively are mediated by the upregulated stress response, increased cellular survival and functional integrity.

Carvedilol pretreatment (i.e., 3 h) was sufficient to induce transient changes in the redox signaling pathways and to mitigate cell death, suggesting the mechanisms involve post-transcriptional activation of the antioxidant defense response and/or post-translational processes for cytoprotection, respectively. Carvedilol appears to possess a modest pro-reducing property capable of modulating the cellular redox threshold in a dose-dependent manner, thereby preventing DOX-induced oxidative stress and the increased likelihood of maintaining ‘redox poise’ (Figure S6). For example, the marked decreases in cellular pools of reduced thiols by DOX treatment, which is associated with an impaired antioxidant defense capacity, are fully rescued to normal levels of total reduced thiols with carvedilol pretreatment, ensuring appropriate amounts of reducing equivalents for antioxidant capacity. Increases in mitochondrial oxidants following DOX treatment were also ameliorated with carvedilol treatment, further demonstrating a redox maintenance effect of carvedilol pretreatment. Notwithstanding, such influences on oxidant levels appear not to mediate imbalances of the antioxidant defense system such as excess reductants that lead to cardiomyocyte death (termed ‘reductive stress’) [67].

At the level of integrated cellular physiology, our studies have rigorously established not only the impairment of mitochondrial bioenergetics by DOX therapy but also that the effects of mitochondrial respiratory parameters could be preserved by carvedilol pretreatment (Figure 6). The ability of the mitochondria to generate ATP in response to energy demands has always served as a reliable hallmark of its functional state and reflects cell viability [68]. Our study has demonstrated that the DOX-induced diminished mitochondrial respiratory function and perturbed energy metabolism [69] can be partially reversed during either prophylactic drug or genetic treatments (Figure 6 and Figure 7), suggesting for the first time that preservation of bioenergetics at the level of mitochondrial OCR is a plausible mechanism for carvedilol’s favorable clinical outcome.

To independently validate the preventative effects of carvedilol for improvements in basal respiration (Figure 6B), maximal respiration (Figure 6C), mitochondrial ATP production (Figure 6D) and spare respiratory capacity (Figure 6E), we examined whether overexpressing PGC-1α in DOX-treated cells would elicit similar functional consequences. Indeed, the mitochondrial functional and metabolic effects on the readout of human iPSC-derived CMs with PGC-1α overexpression partially recapitulated the effects observed for carvedilol pretreatment for the prevention of DOX-induced deficiencies in mitochondrial respiratory function (Figure 7). Therefore, our gene therapy approach to some extent mimicked prior studies of pharmacological activation of PGC-1α to alleviate DOX-induced myocardial injury and mitochondrial damage [4,70] and upregulation of PGC-1α [71], respectively. DOX-induced downregulation of PGC-1α has been demonstrated in other studies [4,70,72] and there are several agents that have been shown to exert cardioprotective effects against DIC via the activation of PGC-1α. The protective effects of PGC-1α activation against DIC have been attributed to PGC-1α-mediated reduction in oxidative stress and mitochondrial protection. Whether carvedilol activates PGC-1α or mediates downstream signaling was not investigated in this study, although it had previously been shown to increase PGC-1α expression and mitochondrial biogenesis in another cell type [73]. While the preservation of mitochondrial function offered by PGC-1α involves its regulation of mitochondria quality control mechanisms [74], carvedilol’s role most likely involves amelioration of mitochondrial oxidants, maintenance of cellular redox balance and oxidative respiratory function.

## Figures and Tables

**Table 1:** List of genes and corresponding forward and reverse primers.

Gene	Forward (5′–3′)	Reverse (5′–3′)
Human Nqo1	CCTGCCATTCTGAAAGGCTGGT	GTGGTGATGGAAAGCACTGCCT
Human HMOX1	CCAGGCAGAGAATGCTGAGTTC	AAGACTGGGCTCTCCTTGTTGC
Human Catalase	GTGCGGAGATTCAACACTGCCA	CGGCAATGTTCTCACACAGACG
Human SOD2	CTGGACAAACCTCAGCCCTAAC	AACCTGAGCCTTGGACACCAAC
Human SOD1	CTCACTCTCAGGAGACCATTGC	CCACAAGCCAAACGACTTCCAG
Human Keap1	CAACTTCGCTGAGCAGATTGGC	TGATGAGGGTCACCAGTTGGCA
Human GAPDH	TCCAAAATCAAGTGGGGCGA	TGATGACCCTTTTGGCTCCC

## Data Availability

The data presented in this study are available on request from the corresponding author.

## Supplementary Materials

The following supporting information can be downloaded at: https://www.mdpi.com/article/10.3390/antiox12081585/s1.
