# Supplementary material for: Carvedilol Phenocopies PGC-1α Overexpression to Alleviate Oxidative Stress, Mitochondrial Dysfunction and Prevent Doxorubicin-Induced Toxicity in Human iPSC-Derived Cardiomyocytes
Source: Antioxidants (Basel). 2023 Aug 9;12(8):1585. doi: 10.3390/antiox12081585 (PMC10451268; doi:10.3390/antiox12081585)
Supplement: Supplementary file 1 [file antioxidants-12-01585-s001.zip › antioxidants-2518165-supplementary.pdf]

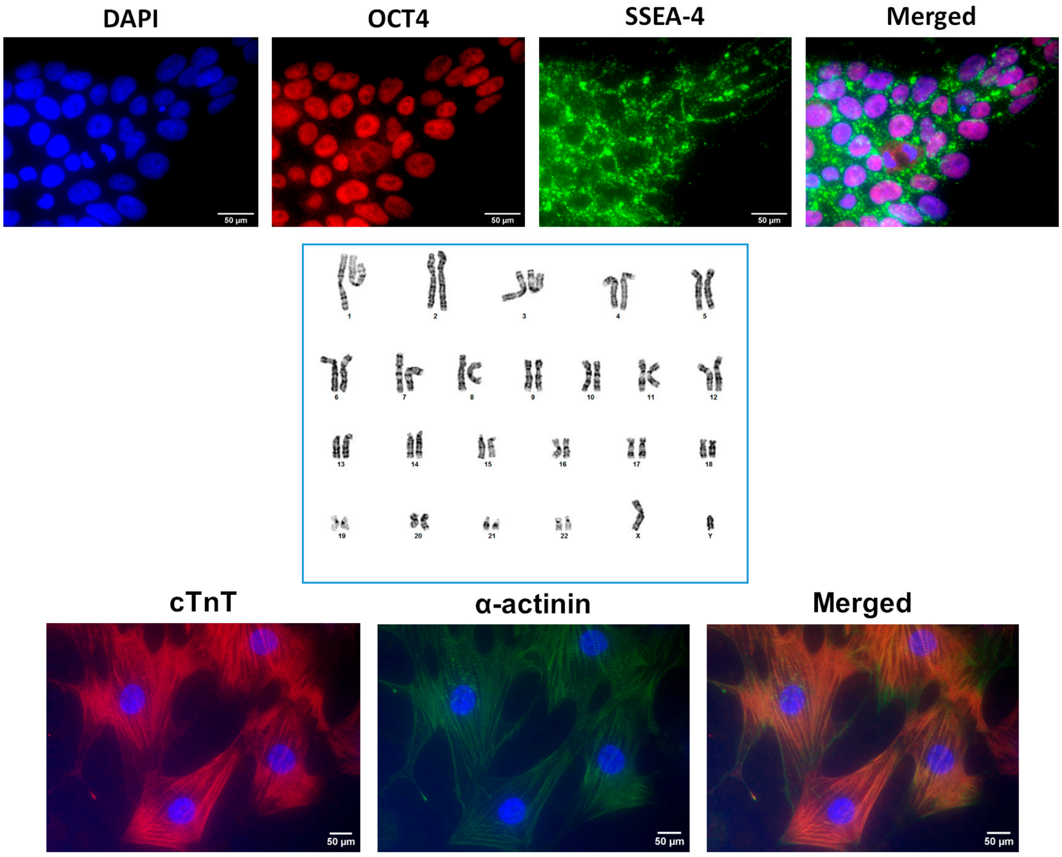

Figure S1. Human iPSC reprograming, karyotyping and cardiomyocyte induction. Representative images of staining for the pluripotency markers SSEA-4 (green) and OCT-4 (red) in iPSCs. Nuclei were counterstained with DAPI (blue). 20 proliferating cells were counted and fully analyzed using G-banding for each cell line, with representative karyotype images showing no consistent abnormalities. Representative images of staining for the markers of mature cardiomyocytes cTnT (red) and a-actinin (green) in iPSC-derived cardiomyocytes (iPSC-CMs).

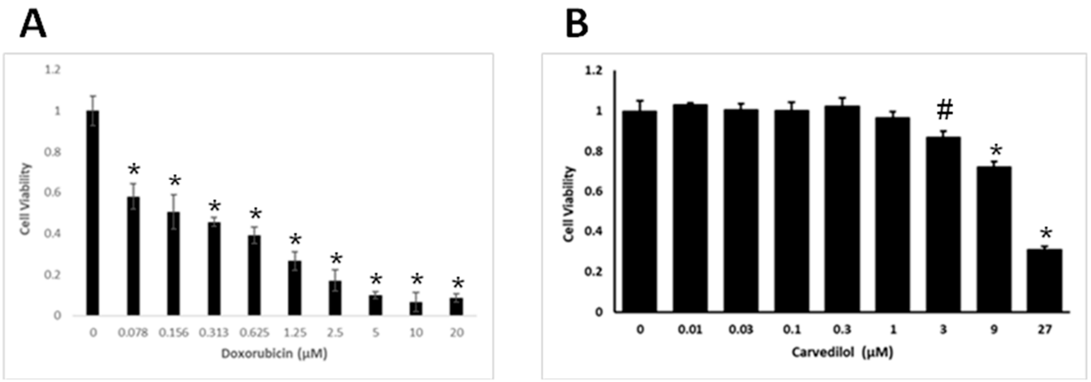

Figure S2. Effect of 48h treatment with DOX (A) and 72h with carvedilol (B) on H9c2 cell viability. Cells were treated with different concentrations of DOX or carvedilol before Alamar Blue assay. Data are normalized to average value of vehicle and presented as mean ±SD. Data are representative of three independent experiments, with each treatment group done in triplicate. (ANOVA; * $p \leq 0.0001$ and # $p \leq 0.001$ vs vehicle group).

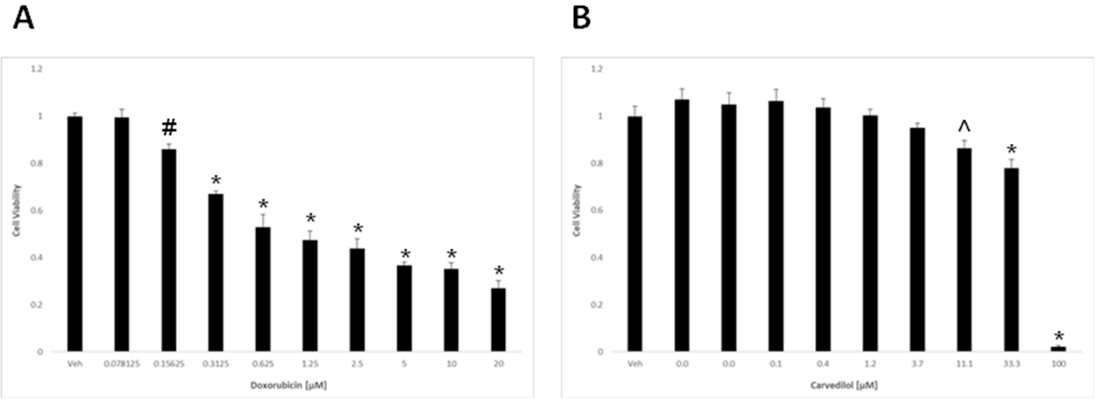

Figure S3. Effect of 48h treatment with DOX (A) and carvedilol (B) on iPSC-CM cell viability. Cells were treated with different concentrations of DOX or carvedilol before Alamar Blue assay. Data are normalized to average value of vehicle and presented as mean ±SD. Data are representative of three independent experiments, with each treatment group done in sextuplicate. (ANOVA; * $p \leq 0.0001$, # $p \leq 0.001$ and ^ $p \leq 0.05$ vs vehicle group).

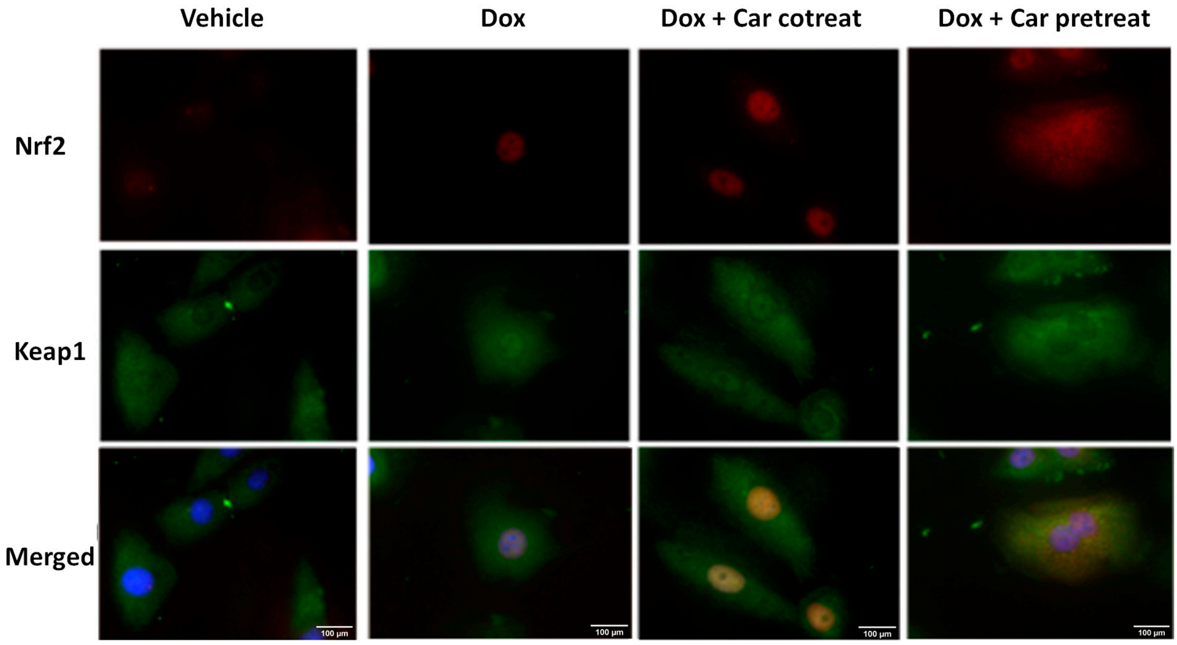

Figure S4. Representative images of Nrf2 and Keap1 co-staining in H9c2 cells following cotreatment or pretreatment with DOX.

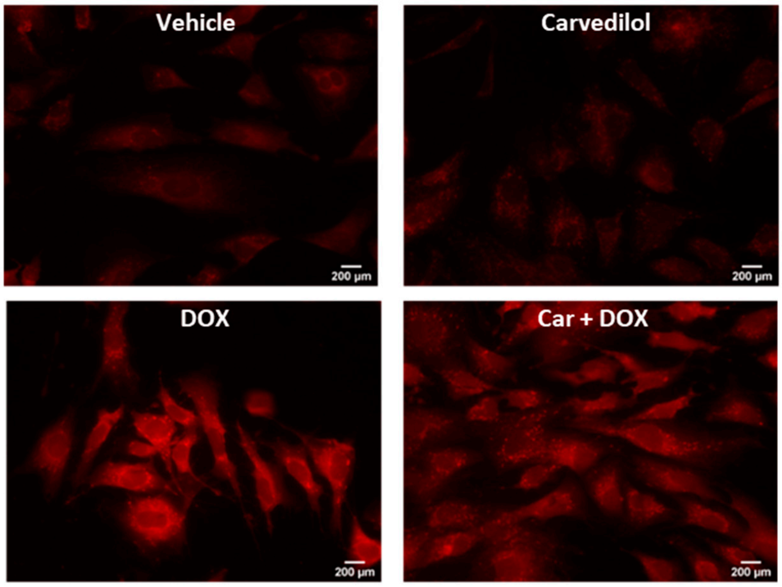

Figure S5. Representative image of MitoSOX assay performed on human iPSC-CMs.

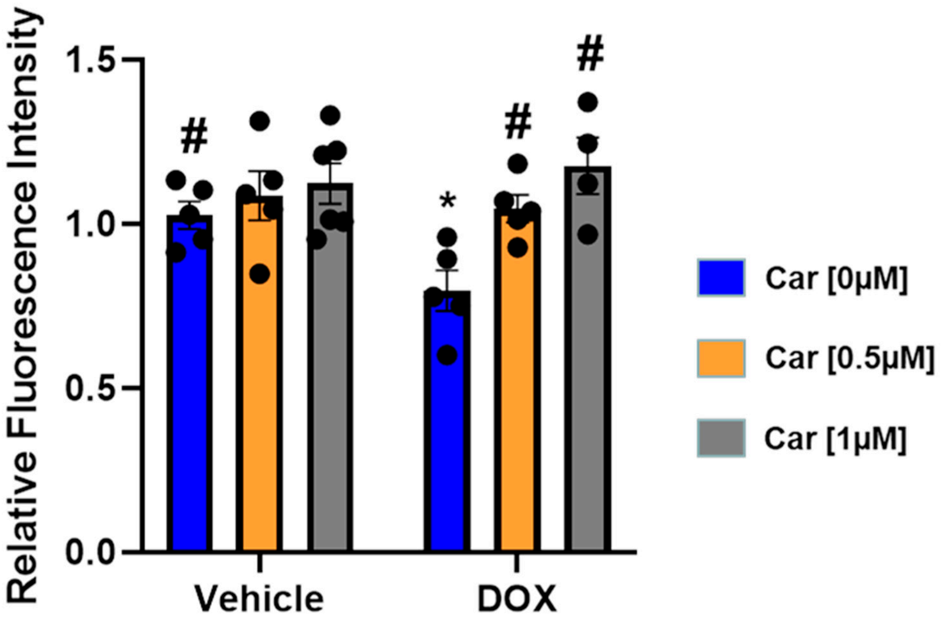

Figure S6. Fluorescence intensities of intracellular total reduced thiols in H9c2 cells following differential treatment. Cells were pretreated for 2h with carvedilol followed by 4h with 0.5μM DOX. Data presented as mean ±SEM of several independent experiments (n = 4–6). (ANOVA; * $p \leq 0.05$ vs vehicle, # $p \leq 0.05$ vs DOX).
